# Dynamic reporters for probing real-time activation of human fibroblasts from single cells to populations

**DOI:** 10.1063/5.0166152

**Published:** 2024-06-24

**Authors:** Samantha E. Cassel, Breanna M. Huntington, Wilfred Chen, Pedro Lei, Stelios T. Andreadis, April M. Kloxin

**Affiliations:** 1 Chemical and Biomolecular Engineering, University of Delaware, Newark, Delaware 19716, USA; 2 Chemical and Biological Engineering, University at Buffalo, Buffalo, New York 14260-4200, USA; 3 Materials Science and Engineering, University of Delaware, Newark, Delaware 19716, USA

## Abstract

Activation of fibroblasts is pivotal for wound healing; however, persistent activation leads to maladaptive processes and is a hallmark of fibrosis, where disease mechanisms are only partially understood. Human *in vitro* model systems complement *in vivo* animal models for both hypothesis testing and drug evaluation to improve the identification of therapeutics relevant to human disease. Despite advances, a challenge remains in understanding the dynamics of human fibroblast responses to complex microenvironment stimuli, motivating the need for more advanced tools to investigate fibrotic mechanisms. This work established approaches for assessing the temporal dynamics of these responses using genetically encoded fluorescent reporters of alpha smooth muscle actin expression, an indicator of fibroblast activation. Specifically, we created a toolset of human lung fibroblast reporter cell lines from different origins (male, female; healthy, idiopathic pulmonary fibrosis) and used three different versions of the reporter with the fluorescent protein modified to exhibit different temporal stabilities, providing temporal resolution of protein expression processes over a range of timescales. Using this toolset, we demonstrated that reporters provide insight into population shifts in response to both mechanical and biochemical cues that are not detectable by traditional end point assessments with differential responses based on cell origin. Furthermore, individual cells can also be tracked over time, with opportunities for comparison to complementary end point measurements. The establishment of this reporter toolset enables dynamic cell investigations that can be translated into more complex synthetic culture environments for elucidating disease mechanisms and evaluating therapeutics for lung fibrosis and other complex biological processes more broadly.

## I. INTRODUCTION

Fibrosis is a class of diseases driven by the activation and persistence of myofibroblasts. While fibroblast differentiation into contractile myofibroblasts is critical for healthy wound healing, persistent activation contributes to excess deposition and cross-linking of collagen and stiffening of tissue and can lead to organ dysfunction and death.^1^ The most common type of pulmonary fibrosis, idiopathic pulmonary fibrosis (IPF), is a disease with uncertain etiology that is responsible for over 50 000 deaths each year in the US alone, where 80% of the patients die within 5 years of diagnosis.^2^ While two Food and Drug Administration (FDA) approved treatments, pirfenidone and nintedanib,^3,4^ modestly slow disease progression, a substantial number have failed in the pre-clinical or clinical pipeline, highlighting a need for additional understanding of disease mechanisms to improve the identification of novel therapeutics. The use of human *in vitro* models of fibrosis complemented with *in vivo* animal models provides a robust approach for hypothesis testing and drug evaluation.^5–10^ However, these approaches currently are limited in their potential to assess the dynamics of human fibroblast responses to complex microenvironment stimuli, requiring additional tools to be developed for probing fibroblast activation in real-time and on a single cell and population basis.

Many current methods utilized to assess fibroblast activation in myofibroblasts focus on characterizing protein expression markers: for example, alpha smooth muscle actin (αSMA) indicative of a mature myofibroblast and contractile cytoskeleton; Yes‐associated protein (YAP) nuclear localization indicative of mechanotransduction and early injury-induced myofibroblastic activation; and collagen I production associated with matrix remodeling by myofibroblasts.^11–14^ However, techniques for measuring these markers are often insufficient or challenging to use for assessing temporal changes, as many are end point assessments and often rely on population averages. Since fibroblast populations are inherently heterogeneous,^15–17^ population averaging techniques, such as reverse transcription quantitative PCR (RT-qPCR) and Western blot, can mask trends in distinct subpopulations. Immunostaining provides single cell resolution; however, this technique is often used as a binary on/off assessment with many protein markers.^18^ Recent advances in cell morphology-based analyses that combine imaging and machine learning methods enable the quantification of the spectrum of myofibroblast activation on a continuous scale, from a “quiescent” fibroblast to a “proto-myofibroblast” intermediate phenotype (positive for diffuse αSMA expression; negative for αSMA-positive stress fibers) and then “mature” myofibroblasts (αSMA-positive stress fibers).^12,14^ While enabling for many investigations, immunostaining does not provide information about the dynamics of active protein transcription or translation, only the presence of translated protein. To improve the temporal resolution of cell assessment, there is a fundamental need for nondestructive methods that allow multiple collections from single samples to expand the investigative toolbox for understanding the dynamics of cell activation in disease initiation and progression.

A few methods exist for examining fibroblast activation using fluorescent reporters, a class of tools where cells are engineered to conditionally express a fluorescent protein, such as in response to the promoter of a protein of interest. One such assay is the luciferase assay; however, this is also population based, end point, and requires large cell counts. Alternatively, lung fibroblasts can be harvested from transgenic animal models engineered to express fluorescent protein when proteins associated with fibroblast activation are expressed (e.g., αSMA, collagen I), providing real-time indicators of protein transcription, although limited to non-human cells.^19^ CRISPR/Cas9 also can be implemented to establish fibroblasts that fluorescently report on αSMA expression with site-specific gene integration, which has been demonstrated with harvested and immortalized cells from Sprague–Dawley rats, showing the utility of genetic modification for creating dynamic reporting tools.^20^ While the opportunities with CRISPR systems are broad and exciting, other techniques exist for gene integration with potentially higher accessibility. For example, lentivirus is a comparable technique for gene incorporation and is attractive for its ability to stably transduce a variety of cell types, imparting random gene integration into the chromosomal DNA for long term stable expression. While random integration can create challenges with senescence that require consideration, lentivirus is versatile for transducing human cell lines as well as primary, patient-derived cell populations, which is critical for the most clinically relevant observations about disease progression and offers opportunities for personalized medicine.^21^ A lentiviral reporter system has been established for reporting on αSMA for characterizing human mesenchymal stem cell (hMSC) differentiation;^22^ however, this system has yet to be applied for studies of fibroblast activation, where timescales differ between different biological processes and transduction varies among cell types.

In this work, we established the use of lentiviral reporters with different temporal stabilities (Stable, Intermediate, and Fast) in human fibroblast populations to track the dynamic expression of the well-known myofibroblast activation marker, αSMA. The varied temporal stabilities enable assessment of transient transcription processes in response to microenvironmental cues for probing the effect of extrinsic and intrinsic factors on the dynamics of fibroblast activation. We established methods for assessing the extent of αSMA expression on a single cell basis, benchmarked vs traditional immunostaining, where population distributions provide insight into subpopulation differences in response to mechanical cues over the course of days. Furthermore, we established imaging methods for assessing cell response to activating cytokines, where we can examine these responses on the timescale of hours, assess dynamic ranges of αSMA expression, and track individual cell fates. Finally, we translated this lentiviral method to primary human lung fibroblasts with a high degree of success. These innovative methods for assessing fibroblast activation dynamics open the door for future mechanistic studies and the development of more effective therapeutics, as well as providing a general approach for the assessment of the expression dynamics of many other proteins.

## II. RESULTS AND DISCUSSION

### A. Approach for reporting on human fibroblast activation processes

**FIG. 1.**
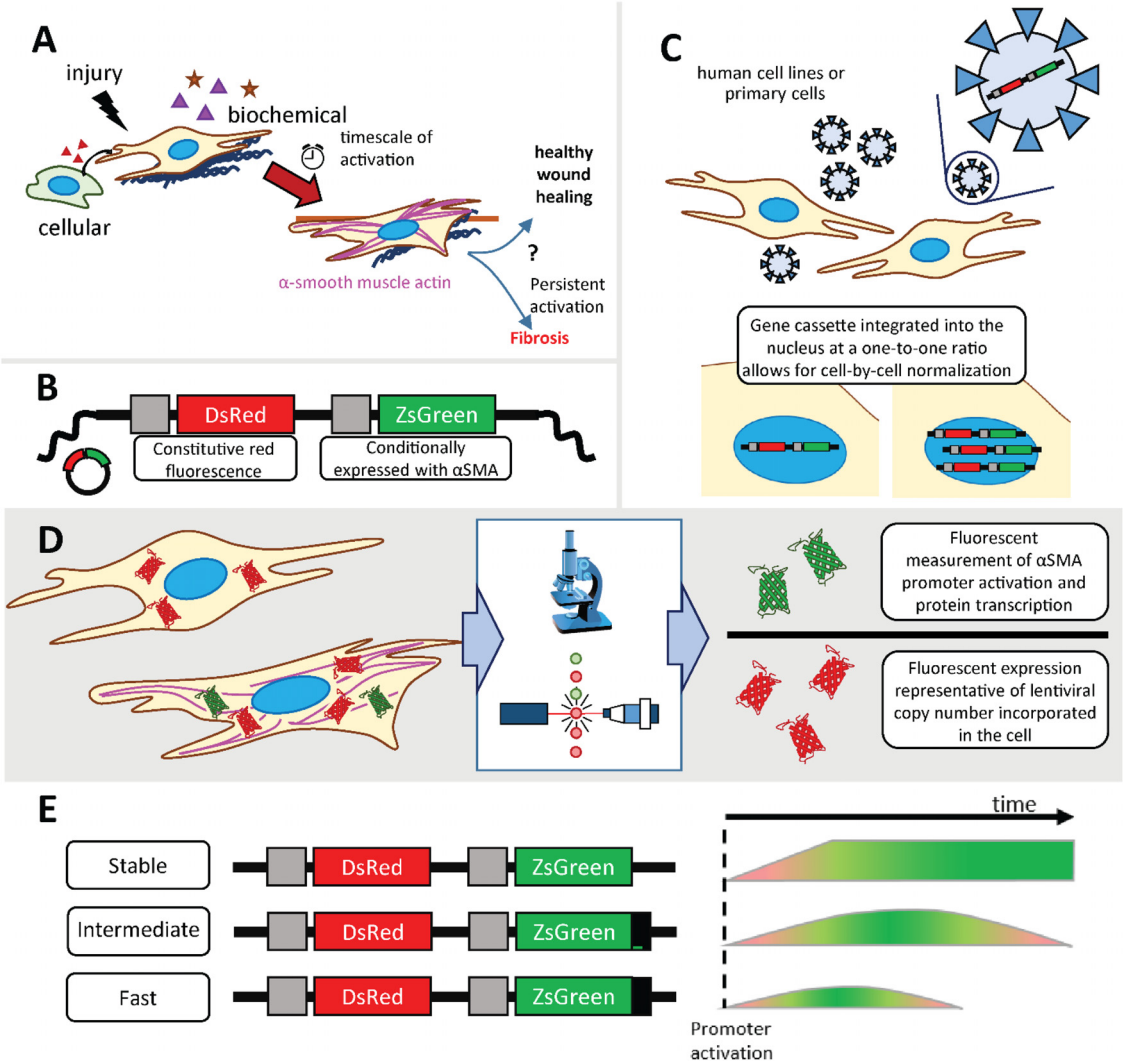
Research strategy. (a) Fibroblast activation is understood to be a result of many microenvironmental cues, such as cell–cell communication, fibrillar structure, biochemical cues, and matrix stiffness, which can either drive healthy wound healing or persistence and fibrosis. There is a need for innovative tools for understanding why certain signals drive homeostasis vs disease. (b) Simplified gene cassettes depict the two key genes for fluorescent reporting: a red fluorescent protein expressed under the constitutive PGK promoter and a green fluorescent protein conditionally expressed with the activation of the ACTA2 promoter associated with alpha smooth muscle actin expression. (c) Fibroblasts are transduced at a high titer to create reporter cell populations, where some cells receive more gene cassette copies than others, but always at a one-to-one ratio of red and green fluorescence. (d) Transduced cells then express constitutive DsRed-Express2 and conditionally express ZsGreen, where the one-to-one ratio of red and green genes delivered to each cell allows for normalization by red fluorescence intensity. (e) To expand the reporter system to track temporal fibroblast behavior, three versions of the gene cassette were utilized, where Intermediate and Fast are modified to degrade on different timescales.

Fibroblast activation is triggered by a myriad of microenvironmental cues, for example, biochemical signals like TGFβ1, mechanical cues like matrix stiffness or fibrillar structure, or intracellular interactions (e.g., other fibroblasts, epithelial cells, or immune cell populations) [Fig. 1(a)]. The dynamics of fibroblast responses to these cues are thought to play a critical role in homeostasis or the initiation and progression of disease after injury. Innovative tools are needed for probing this complex process within human *in vitro* models for insight into fundamental disease mechanisms and the evaluation of new therapeutic approaches. To address this need, we established a range of human lung fibroblast reporters for probing the dynamics of activation in response to different microenvironment cues. Cell lines were created using lentivirus for transduction of fibroblasts from different origins (male, female; healthy, IPF) and reporting on αSMA expression on different timescales using ZsGreen with different stabilities (Stable, Intermediate, and Fast). Techniques were established for analyzing expression on a single cell and population basis and in real time, using confocal imaging, image analysis software (Imaris, Oxford Instruments), and data transformation and statistical methods. Furthermore, this approach was applied to primary human lung fibroblasts to demonstrate applicability for more clinically relevant investigations and personalized medicine.

The lentiviral vector design utilizes two fluorescent proteins under separate promoters. First, a ZsGreen fluorescent protein is expressed under the ACTA2 promoter for αSMA, a cytoskeletal protein implicated in the contractile phenotype of differentiated myofibroblasts and a marker of fibroblast activation. This ZsGreen expression allows for the real-time monitoring of αSMA expression in response to microenvironmental cues. A second fluorescent protein, discosoma red fluorescent protein (DsRed)-Express2, is expressed under a constitutive human phosphoglycerate kinase (PGK) promoter and serves as a marker of successful lentiviral transduction [Fig. 1(b)], where cells transduced with this construct are only capable of expressing ZsGreen if they are also expressing DsRed (Fig. S1). Since the genes for both fluorescent proteins are within the same lentiviral transfer vector, the genes are incorporated into the cell's genome at a one-to-one ratio [Fig. 1(c)], and the DsRed intensity can be used to normalize αSMA-associated ZsGreen expression for quantification independent of gene transfer efficiency [Fig. 1(d)].^22^ To ensure that the delivery of lentivirus at a high titer did not impact cell function broadly, metabolic activity was compared between transduced and non-transduced cell types, with no distinguishable difference in behavior (Fig. S2); specifically, consistent and increasing metabolic activity was observed over time in all conditions, and no statistical difference in metabolic activity was observed between transduced and non-transduced conditions, suggesting cell health/proliferation were maintained upon transduction and that senescence broadly was not induced with this treatment. This observation is consistent with prior published work of the Andreadis lab using these and similar lentiviral reporters: transduced cells exhibited retention of cellular functions, including proliferation, response to stimuli, and differentiation capacity for a range of cell types, including epidermal keratinocytes, bone marrow mesenchymal stem cells, and hair follicle stem cells.^22–25^

These lentiviral αSMA reporter systems include (i) a reporter with stable ZsGreen protein (from here on referred to as “Stable”) and (ii) a reporter ZsGreen modified by a degradation tag (commercially available ZsG-DR; from here on referred to as “Fast”) [Fig. 1(e)], which were established by the Andreadis lab to investigate the impact of a library of silencing ribonucleic acid (RNA) on the differentiation of human mesenchymal stem cells (hMSCs) by reporting expression of αSMA.^22^ To complement the “Stable” and “Fast” reporters for probing fibroblast activation, in this work, we created a second destabilized reporter with a longer half-life (from here on referred to as “Intermediate”). Specifically, the new “Intermediate” reporter was designed with four amino acid substitutions within the degradation tag (Fig. S3) that have been demonstrated in Chinese hamster ovary (CHO) cells to increase the half-life by approximately four times in the magnitude relative to ZsG-DR.^26^ In our assessments, the half-life of the Intermediate reporter is just over an hour (t_1/2_ = 1.33 ± 0.52 h, Fig. S4, Table S1),^27,28^ and the Fast reporter qualitatively degrades more rapidly than the Intermediate although its short half-life is not quantifiable with the imaging methods used (Fig. S5). All three lentiviral reporter systems (Stable, Intermediate, and Fast) were then applied to human lung fibroblasts from different backgrounds for their stable transduction: specifically, two were derived from healthy human lung tissue (CCL151, male; CCL210, female) and one was derived from IPF human lung tissue (CCL134, female).

With this expanded toolset, we can build upon the previously established reporter approach to investigate fibrotic cellular mechanisms with additional timescales and gain insight into the duration of protein transcription processes. By expanding the approach to look at cells on an individual basis, we also can gather information about the differential response of cell subpopulations, including differences among fibroblasts from different origins (e.g., healthy vs diseased tissues). Thus, in this contribution, we aimed to establish relevant methods and apply this system for assessing the activation behavior of fibroblasts in response to different microenvironment cues with single cell and temporal resolution and probe the dynamics of cell behavior across a range of human cell sources.

### B. Stable reporter correlates with traditional immunostaining techniques

**FIG. 2.**
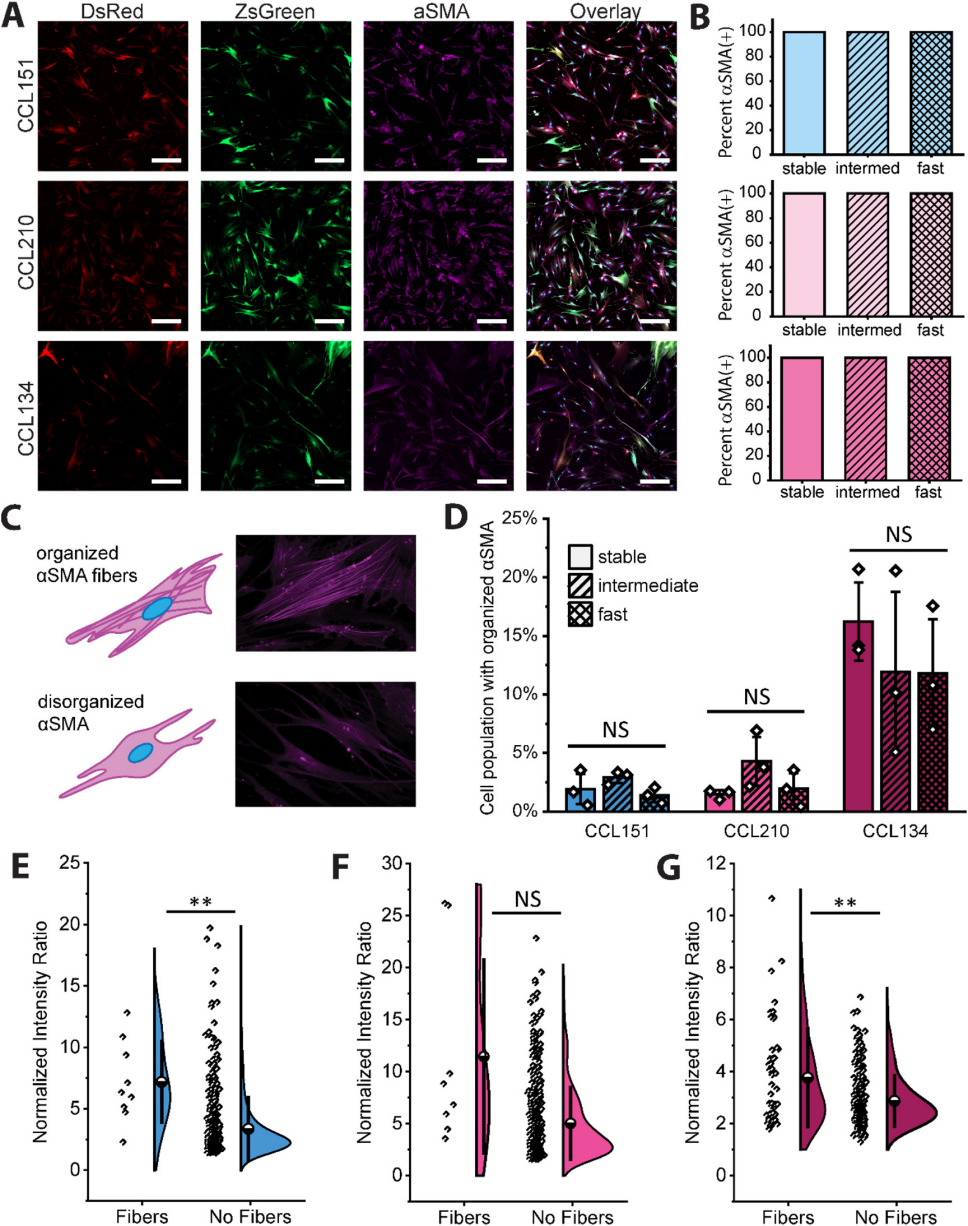
Comparison of αSMA expression with immunostaining assessment. (a) Stable reporter lung fibroblasts (CCL151, male healthy; CCL210, female healthy; and CCL134, female IPF) were cultured on glass for 48 h, fixed, and stained for alpha smooth muscle actin. (b) Quantification of immunostained images demonstrates that samples from all reporter lines (Stable, Intermediate, and Fast) of all cell types had 100% of the cells positive for diffuse staining for alpha smooth muscle actin (n = 3 wells per condition, >50 cells per well). (c) To further differentiate proto-myofibroblasts from mature myofibroblasts, cells with organized αSMA fibers (CCL151-stable reporter) were visually assessed and (d) quantified across all reporter cell lines (n = 3 well per condition, >50 cells per well). (e)–(g) Reporter intensity normalized on a per cell basis was compared for cells with and without organized αSMA fibers for CCL151 (e), CCL210 (f), and CCL134 (g) cell lines. (per cell line, data from all wells were pooled for distributions). Scale bars are 200 *μ*m. Statistical comparisons in (d) were assessed by one-way ANOVA and comparisons in (e)–(g) were assessed by Welch's t-test. ^*^p < 0.05, ^**^p < 0.01, ^***^p < 0.001, and ^****^p < 0.0001.

To first assess the utility of active fluorescent reporting on fibroblast activation, we wanted to compare it with traditional methods for examining cell activation on both a single cell and a population basis, such as immunostaining of fixed cells for αSMA. The cell lines stably transduced with “Stable,” “Intermediate,” and “Fast” reporters were cultured on glass for 48 h and assessed for αSMA expression by immunostaining. For all cell types in all reporter conditions, approximately 100% of the population stained positive for diffuse αSMA [Figs. 2(a) and 2(b)]. Considering these are cell lines with a history of propagation on hyperphysiological substrates [i.e., tissue culture polystyrene (TCPS)], this result was unsurprising, as fibroblasts are well known for their mechanical memory.^29^ Despite this, immunostaining can provide further distinction of subpopulations by identifying stress fiber formation [Fig. 2(c)]. As fibroblasts activate, they first undergo a phenotype often referred to as proto-myofibroblasts, where αSMA is expressed and present, but diffuse within the cytoplasm. As these proto-myofibroblasts mature, the αSMA assembles into stress fibers; consequently, IPF is characterized by a large population of persistent, mature myofibroblasts, indicated by robust stress fiber formation.^16^ Indeed, the IPF derived cell line (CCL134) showed a markedly higher population fraction with identifiable stress fibers (12%–15%) across all three reporters, whereas the healthy fibroblast cell lines (CCL151 and CCL210) showed percentages closer to 2%–5% [Fig. 2(d)]. Of note, these data can be utilized as a secondary check of cell function after transduction. There are no statistical differences in stress fiber cell counts between reporters within each cell type, indicating that their response to a hyperphysiological substrate modulus stimulus is consistent across three different lentivirus treatments. To assess the utility of reporter function compared with immunostaining approaches, we then investigated whether there was correlation between mature myofibroblast percentage and higher normalized fluorescence intensity from reporter quantification. Normalized fluorescence intensity was measured from the same immunostained fluorescence images and correlated with stress fiber identification. Note that all DsRed(+) cells demonstrated a mean ZsGreen fluorescence intensity above the background baseline, so all DsRed cell objects identified in the analysis were included in the plotted distributions.

Within each cell type, cells were divided into two subpopulations, stress fibers and no stress fibers, and the distributions of normalized intensity ratio for each were plotted for comparison. Results for the Stable reporter [Figs. 2(e)–2(g)] show that, for male healthy fibroblasts (CCL151) and IPF derived fibroblasts (CCL134), stress fiber populations have a significantly higher normalized intensity when compared with cells lacking stress fibers. Furthermore, in the case of female healthy fibroblasts (CCL210), there is a qualitative increase in the distribution mean, though the p-value of this assessment is 0.09 (Table S4). When comparing these subpopulations for the Intermediate (Fig. S6) and Fast reporters (Fig. S7), there were limited differences between the stress fiber and no stress fiber subpopulations (Table S4), indicating that the presence of fibers themselves is not necessarily indicative of differences in the rate and extent of transient expression at a detectable level.

Overall and importantly, the Stable reporter results suggest that dynamic reporting of αSMA protein transcription correlates with protein expression and stress fiber formation differences. Given the reporter system design, it is reasonable that the Stable reporter identifies these differences, as the Stable reporter provides information about long term expression behavior. The more the αSMA promoter is activated and the protein expressed, more ZsGreen accumulates in the cytoplasm and is not turned over at a rapid rate. Therefore, cells with mature stress fiber formation would have a more substantial history of ZsGreen expression and a higher reporting intensity. Notably, these results emphasize that the Stable reporter has utility for investigating the progression of more mature myofibroblast subpopulations, and the reporter toolset in total provides complementary insights to immunostaining techniques when investigating end point conditions.

When interpreting the normalized fluorescence distributions, it is most effective to directly compare responses between conditions within the same cell source (e.g., CCL134 between fibers and no fibers, between cell densities, or between substrate stiffnesses). Comparison of absolute values of normalized intensity between cell sources (e.g., CCL151 vs CCL134) may be less reliable due to potential intrinsic differences between cell line metabolic rates and protein turnover rates, though general trends can be elucidated and used for further qualitative understanding. For example, while CCL134 normalized intensities are lower than CCL151 normalized intensities in Figs. 2(g) and 2(e), respectively, the trends within each cell line demonstrate that stress fiber-positive fibroblasts correlate with a higher reporter normalized intensity and that this trend persists across all cell lines tested, though to different extents.

### C. Cell density and origin influences αSMA expression dynamics

This reporter tool provides opportunities for examining snapshots of transient expression behavior for single cells and populations with end point assessments such as flow cytometry. Instead of looking at normalized intensity ratio distributions, one can assess the population fraction expressing ZsGreen above a control cell population's baseline. Flow cytometry gating procedures can distinguish on/off populations more precisely than imaging-based approaches and with higher throughput, providing a complementary tool that is not dependent on microscope system configurations. Identifying this ZsGreen(+) population fraction provides information about active or recent αSMA transcription, providing additional information about differences in the expression between cell populations that is not detectable by immunostaining. With immunostaining, as seen in Fig. 2(b), all cells in these human fibroblast lines that have been propagated on tissue culture plastic, whether originally from healthy or diseased tissue, exhibit diffuse αSMA. Utilizing the different timescale reporters, subpopulations become detectable with the Intermediate and Fast reporters, while the Stable reporter correlates with the αSMA immunostaining result, all together complementing and providing additional information beyond the binary output of immunostaining as detailed below.

Here, threshold intensities for both ZsGreen and DsRed were determined from a non-transduced control to isolate populations of positively transduced (DsRed positive) cells. From these, the subpopulation also expressing ZsGreen was identified and considered. For the Stable reporter, this fraction represents the cells that have expressed αSMA protein within a long memory (days), whereas the Intermediate and Fast reporters will reflect a population fraction of cells actively expressing protein in the order of hours.

**FIG. 3.**
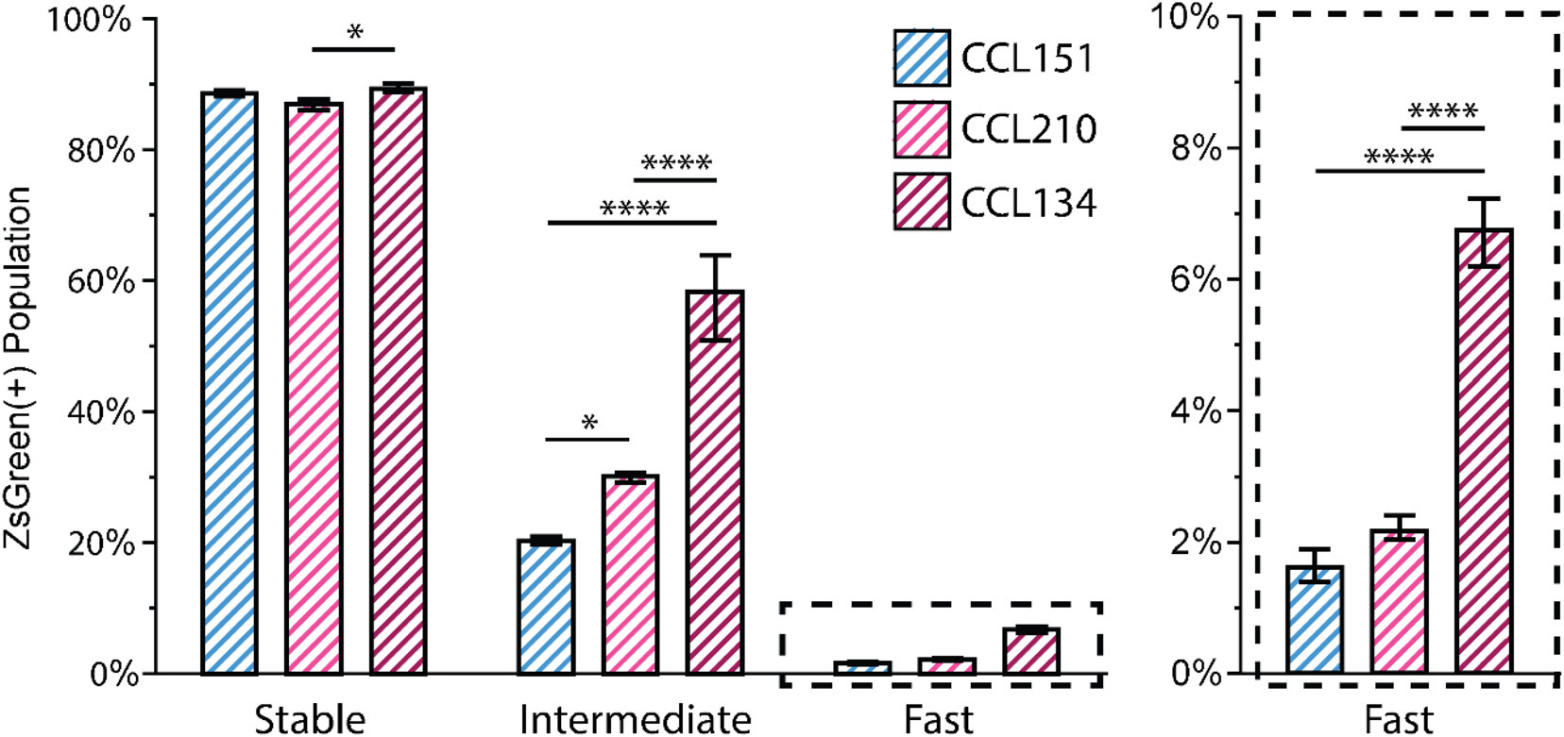
Comparison of reporters between cells of different tissue origin. ZsGreen(+) population fractions for CCL151 (male healthy lung fibroblasts), CCL210 (female healthy lung fibroblasts), and CCL134 (female IPF lung fibroblasts) Stable, Intermediate, and Fast reporters after 48 h culture were assessed by flow cytometry (initial seeding density 5000 cell/cm^2^). Statistical differences were determined within reporters by one-way ANOVA. Error bars represent standard deviation, n ≥ 3 replicates. ^*^p < 0.05, ^**^p < 0.01, ^***^p < 0.001, and ^****^p < 0.0001.

We first characterized a snapshot of reporting after culturing all reporters of all cell lines on TCPS for 48 h and then characterizing them with flow cytometry. Across the three cell lines, the ZsGreen(+) population fraction in the Stable reporter is similar, where the only statistical difference seen is between CCL210 (healthy fibroblasts) and CCL134 (IPF fibroblasts). This limited variation within the Stable reporter was expected given the immunostaining results. However, in the Intermediate and Fast reporters, there is a notable increase in population fraction exhibiting ZsGreen fluorescence for the IPF derived lung fibroblasts (CCL134) in comparison to normal lung derived fibroblasts (CCL151 and CCL210) (Fig. 3, Table S5). This observation could be indicative of transcriptional differences between diseased and healthy lung fibroblasts, influenced by long term exposure to diseased tissue or other intrinsic differences in cell response.^29^

To further examine what intrinsic differences may be elucidated by the reporter, we wanted to investigate how culture density impacted activation of fibroblasts from different origins. Activated myofibroblasts in IPF are found in structurally distinct regions called fibroblastic foci, where a compacted accumulation of contractile myofibroblasts forms and is surrounded by acellular regions of a stiff, hyper-crosslinked collagen matrix. Investigations in the literature have hypothesized that cell density and extracellular matrix (ECM) confinement contribute to myofibroblast activation and persistence of an activated, fibrotic phenotype.^30^ For example, primary human lung fibroblasts (HLFs) cultured at varying densities and stimulated with TGFβ1 exhibited αSMA expression that was highly dependent on cell density, with HLFs in higher density cultures having significantly increased αSMA expression upon exposure to TGFβ1 than in lower density cultures. However, this trend varies substantially across the literature, with fibroblasts sourced from other tissues showing opposite trends, and time in culture and proliferation rate seem to also impact response.^31–33^

Most of these investigations are conducted on primary cells, where mechanical memory from culture on hyperphysiological substrates is minimized and differences between αSMA(−) and αSMA(+) populations are more distinct. For the reporter cell lines, we wanted to investigate whether dynamic reporting was able to distinguish protein transcription variation between different culture densities across cells sourced from both healthy and diseased tissue. To do this, all cell lines of all three reporter types were seeded at 5000 cells/cm^2^, 10 000 cells/cm^2^, and 20 000 cells/cm^2^ and cultured for 48 h. Additionally, cells seeded at 5000 cells/cm^2^ were cultured for 96 h to confluence to 1) investigate differences in expression over time with the same starting density and 2) compare reporting results of samples with similar final densities (20 000 cells/cm^2^ for 48 h vs 5000 cells/cm^2^ for 96 h).

**FIG. 4.**
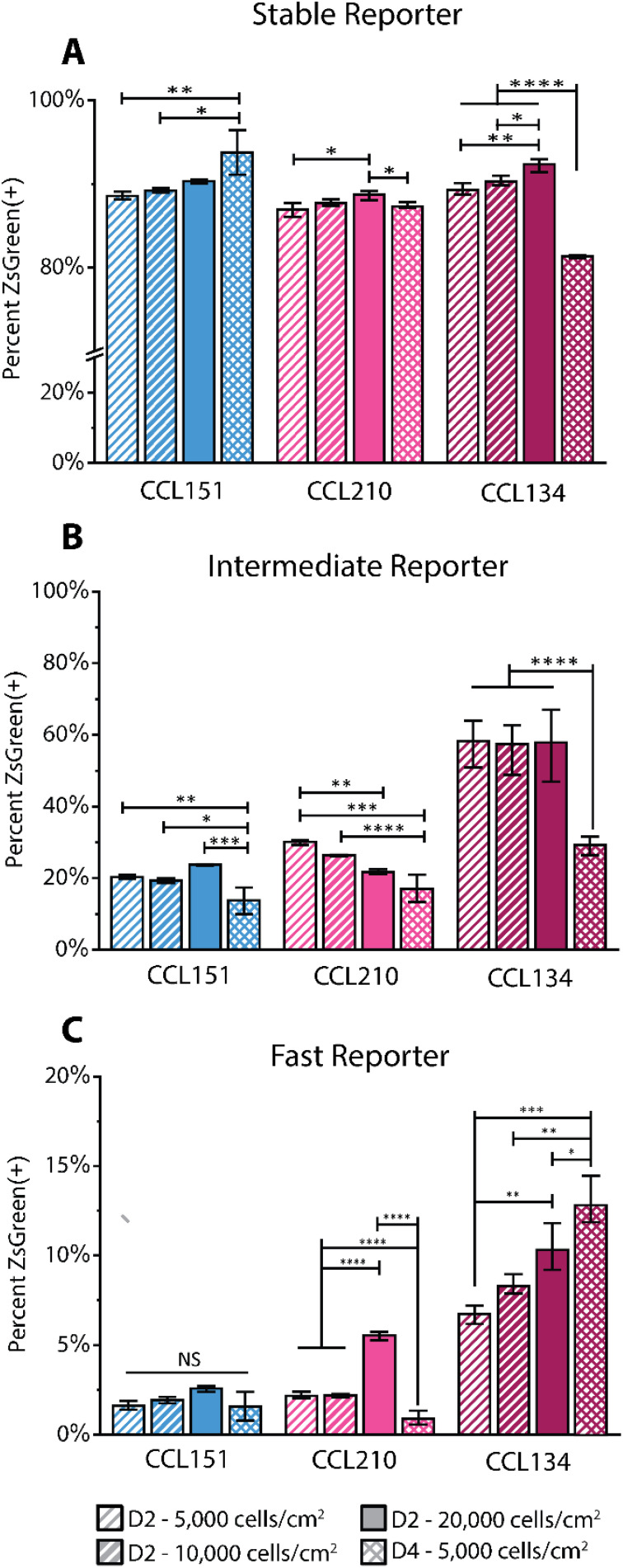
Impact of culture density on the reporting of fibroblast activation. CCL151, CL210, and CCL134 lines at different initial seeding densities and culture timepoints were investigated by flow cytometry with (a) Stable, (b) Intermediate, and (c) Fast reporters. Data for 5000 cells/cm^2^ 48 h conditions are repeated here from Fig. 3 for ease of comparison. Statistical differences were assessed by one-way ANOVA within each cell line and reporter. Error bars depict standard deviation with n ≥ 3 replicates. ^*^p < 0.05, ^**^p < 0.01, ^***^p < 0.001, and ^****^p < 0.0001.

We observed small differences in the ZsGreen(+) population fraction in the Stable reporter conditions, with a significant population of activated cells. Among the 48 h timepoints, there is a slight statistical increase in the ZsGreen(+) fraction as seeding density increases [Fig. 4(a), Table S6]. When comparing the 20 000 cells/cm^2^ 48 h and 5000 cell/cm^2^ 96 h conditions, where final densities are similar, we see differing trends across the cell types. For CCL151, ZsGreen(+) population continues to increase, whereas in CCL210, the fraction is lower at the 96 h timepoint. This suggests that, even across cell populations collected from healthy tissue samples from the same organ system, there can be intrinsic differences in response to cell density and culture time. In the CCL134 IPF cells, this fraction drops significantly, which could indicate that within these diseased-tissue sourced fibroblasts, there is a subpopulation, in response to cell density and culture time, whose phenotypic protein expression shifts away from αSMA.

In the Intermediate and Fast reporter conditions, the IPF derived cell line has a larger subpopulation expressing a baseline level of αSMA than the healthy lung fibroblasts across all densities tested [Figs. 4(b) and 4(c); Tables S7 and S8]. Notably, in both the Intermediate and Fast reporters, the IPF cell population positive for αSMA-associated ZsGreen expression is approximately three times the healthy population. More generally, across most transient conditions, ZsGreen(+) population increases with density, suggesting more activation in response to higher density cultures when controlled for time in culture. Interestingly, in the IPF derived lung fibroblast Intermediate reporter, the 5000 cells/cm^2^ cultured for 4 days has a much lower fraction of ZsGreen(+) cells than the 20 000 cells/cm^2^ density cultured for 2 days, even though these two seeding conditions generally ended at similar cell densities (Fig. S8). However, this trend is opposite in the Fast reporter for the IPF lung fibroblasts. At day 4, this decrease in Intermediate ZsGreen(+) fraction but increase in the Fast ZsGreen(+) fraction might suggest that there is a small subset of cells with high rates of αSMA transcription that can be differentially identified by the Fast reporter, but is masked by the longer half-life of the Intermediate.

### D. Cell culture substrate mechanical properties influence αSMA expression dynamics

**FIG. 5.**
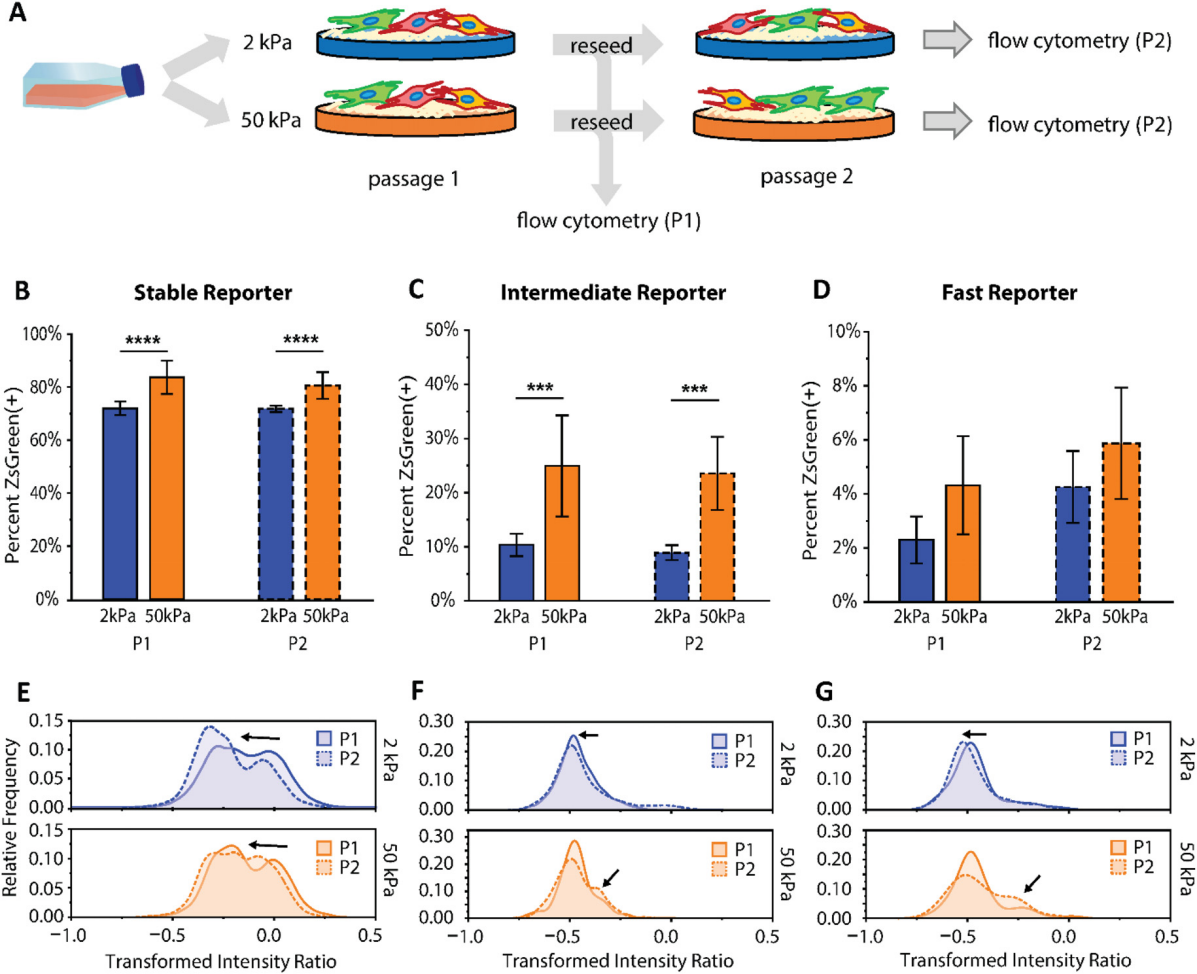
Mechanical properties of the culture substrate impact extent of fibroblast activation. (a) Workflow for assessing the impact of culture surface mechanical properties on αSMA reporting behavior; CCL151 fibroblasts of all reporter types (Stable, Intermediate, and Fast) were cultured on surfaces with different stiffness and assessed using flow cytometry. Error bars represent standard deviation of n ≥ 3 replicates. (b) Stable, (c) Intermediate, and (d) Fast reporter ZsGreen(+) population fractions after culture on soft (E∼2 kPa) and stiff (E∼50 kPa) substrates over two passages. (e) Stable, (f) Intermediate, and (g) Fast box-cox transformed (λ = 0.075) distributions of normalized reporter intensities for individual cell events, where arrows point to shifts in population from P1 to P2. Statistical analysis in panels (b)–(d) were assessed by two-way ANOVA, with passage and culture substrate as factors. ^*^p < 0.05, ^**^p < 0.01, ^***^p < 0.001, and ^****^p < 0.0001.

To investigate the utility of the reporters for characterizing differential cell responses to biomechanical cues, reporter cells were transitioned to culture substrates with moduli representative of native tissue, as fibroblasts in two-dimensional culture are known to preferentially activate on stiff substrates.^6,34,35^ Specifically, we cultured CCL151 healthy lung fibroblasts of the three reporter types on polyacrylamide hydrogels coated with rat tail collagen I with Young's moduli of (1) 2 kPa (in the range of healthy lung tissue) and (2) 50 kPa (in the range of fibrotic lung tissue) [Fig. 5(a)]. The cells were seeded at 5000 cells/cm^2^ on each surface and allowed to approach confluence over 4 days in culture. After 4 days, the cells were propagated on the same surface conditions for a second passage to assess the consistency and longevity of the cell response to each environment. At each passage, cell fluorescence intensity was quantified using flow cytometry.

First, we quantified the data by ZsGreen(+) population fraction to track the differences in αSMA transcription after cells were introduced to these new mechanical stimuli. Looking at the Stable reporter, the 2 kPa substrate demonstrated a significantly lower population exhibiting ZsGreen fluorescence, which persisted over both passages, demonstrating that there are important differences in αSMA expression between 50 kPa “fibrotic” stiffness and 2 kPa “healthy” stiffness that can be detected even with this long timescale reporter. This trend also can be seen using cells transduced with both the Intermediate and Fast constructs [Figs. 5(c) and 5(d); Tables S10 and S11]. Since the 50 kPa polyacrylamide mimics the mechanical properties of “fibrotic” tissue, this response results in a persistence of higher transient αSMA expression activity.

When considering the normalized intensity of each cell plotted in a population distribution, differences in activation response can be seen that are not apparent in the ZsGreen(+) cell fraction comparisons [Figs. 5(e)–5(g)]. For investigating distributions of normalized intensity, raw intensity values for both fluorescent proteins were exported from the flow cytometry data and used to calculate a normalized intensity ratio for each cell, where only ZsGreen(+) cells were considered. These datasets were plotted as distributions for relative comparisons between both substrates and passages. Here, a box-cox transformation was applied to all datasets to reduce data skew and improve interpretation of relative population shifts.

Interestingly, the Stable reporter shows distribution width differences between substrate conditions, with the widest variation in intensities in the 2 kPa condition, suggesting increased heterogeneity [Fig. 5(e)]. Additionally, the 2 kPa distributions are slightly shifted left (indicated by black arrows), consistent with a lower extent of activation-associated protein expression. There are also significant distribution shifts to the left between passages 1 and 2, in both the 2 and 50 kPa conditions, despite the population fractions being the same. Notably, the left mode in the 2 kPa bimodal distribution is significantly larger at passage 2. It is known that culturing fibroblasts on soft substrates decreases activation, and to a lesser extent can decrease mechanical memory, and the leftward shift that continues from passages 1 to 2 indicates that the reporter can provide temporal tracking of these phenotypic changes. This leftward shift is present to a lesser extent for the 50 kPa modulus, suggesting that transitioning from TCPS to the 50 kPa substrate influenced the activation behavior of the cells, but at a slower rate than the 2 kPa “healthy” tissue modulus. Overall, these observations could suggest a lessening of myofibroblast phenotype with culture on physiologically relevant moduli that is observable with the reporter system. Previous work has shown that fibroblasts can de-activate from myofibroblasts to quiescent fibroblasts when transitioned from stiff substrates to soft substrates.^6,34^ The Stable reporter provides opportunities for examining these types of phenomena.

The normalized intensities for the Intermediate and Fast reporters are expectedly much lower than the Stable reporter, due to the more rapid turnover of the destabilized versions of the ZsGreen protein. Therefore, subtle shifts in the distribution could be indicative of changes in subpopulation responses to the microenvironment. With these more rapid reporters, in the 2 kPa condition, we observe a trend of distributions shifting slightly left [Figs. 5(f) and 5(g)], indicating to a lesser extent a tendency toward a less activated phenotype. Interestingly, in the 50 kPa condition, we observe a subpopulation grow as a right-side mode (indicated by black arrows), indicating a subpopulation of cells with increased αSMA expression activity after a second passage on the fibrotic-like stiffness condition. These observations provide a secondary indication of differential cell response and protein transcription behavior on 50 kPa culture substrates when compared with soft 2 kPa. Pairing both population fractions and population distribution analyses provides a robust demonstration of the broad utility of the reporters, both Stable and more rapid Intermediate and Fast, to assess dynamic differences in cell response to various microenvironmental cues.

### E. Reporter system aids in tracking single cell protein expression in response to biochemical stimuli

**FIG. 6.**
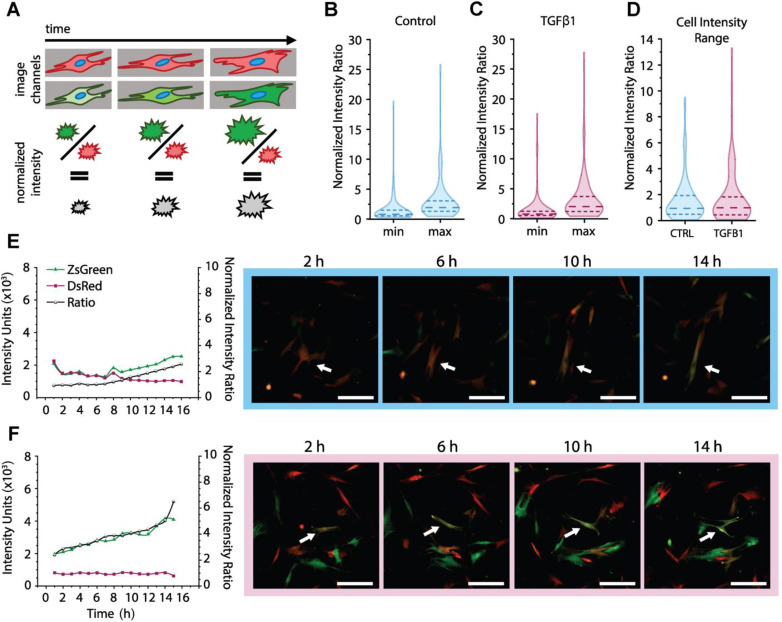
Temporal dynamics of activation in response to biochemical cues: Stable reporter. (a) Timecourse image studies identified and quantified individual cell fluorescence intensity values over time and calculated normalized intensity ratios for single cells at each timepoint. (b) Distribution of the minimum and maximum normalized intensity value of each cell over the timecourse (16 h) for control cells. Here, the distributions are represented with violin plots, where the width of the curve corresponds with the frequency of data points, the long dashed line notes the mean, and the short dashed lines note quartiles. (c) Distribution of the minimum and maximum normalized intensity value of each cell over the timecourse for TGFβ1 treated cells. (d) Distribution of normalized intensity range per cell over the timecourse for both control and TGFβ1 treated cells. For each violin plot, >80 cell tracks were analyzed. (e)–(f) Individual cell tracks of DsRed, ZsGreen, and normalized intensity ratio values over the timecourse from the (e) control cell population, and (f) the TGFβ1 treated population. Scale bars are 25 *μ*m. White arrows depict the tracked cell body.

We can expand the use of this reporter system even further to not only track changes in populations at time points of interest but to also track individual cell expression over time through real-time imaging analysis. While the effects of microenvironmental cues such as modulus and protein structural elements can be examined in the order of hours to days, there are other cues (e.g., soluble factors) with signaling on a shorter timescale that can be more challenging to assess with that limited temporal resolution. Therefore, our ability to image live cells and capture their fluorescence intensities over time is critical for characterizing responses to various soluble biochemical cues, such as response to TGFβ1. To establish a method for assessing these shorter timescale responses, we chose to assess the fibroblast response to TGFβ1 by fluorescence microscopy. In this study, we seeded both Stable and Intermediate CCL151 lung fibroblasts for 24 h before treating them with 10 ng/mL of TGFβ1. We then captured their response by fluorescence microscopy over the 16 h immediately after treatment to track changes in transcriptional processes of αSMA expression [Fig. 6(a)].

We first looked at Stable reporters and investigated the shift in normalized intensity distribution at each timepoint (Fig. S9), finding that the TGFβ1 condition had slightly higher normalized values than the control over time. Due to the heterogeneity of the cell population, we then investigated the dynamic reporting range of normalized intensities for each individual cell over the timecourse for both the control and TGFβ1 conditions. When comparing the distributions of minimum and maximum intensities for control and for treatment [Figs. 6(b) and 6(c); Table S12], the maximum for the TGFβ1 condition has a wider and longer upper tail, suggesting a larger population fraction is expressing higher normalized intensity values and has a larger increase in αSMA expression. While not statistical, this trend is further visible when we consider the relative dynamic range of the normalized fluorescent intensity for each cell [Fig. 6(d)]. As shown in Fig. 6(d), when the normalized fluorescent intensity for individual cells is plotted as distributions for both control and treatment, the TGFβ1 condition again shows a longer and broader upper tail, demonstrating a potential subpopulation shift that is not well represented by assessment of the means (long dashed lines) or quartiles (short dashed lines).

This imaging technique allows for tracking of the fluorescence of individual cells, which we call “cell tracks,” as shown in Figs. 6(e) and 6(f) and additional traces in Fig. S10. For each cell object tracked, as indicated by white arrows in the images, the values for DsRed and ZsGreen fluorescence intensity are plotted over time, as well as the normalized intensity. Most cell tracks with dynamic value changes show only an upward trend in this normalized intensity value over this timeframe, consistent with the Stable reporter system design. A representative track for the control cell population is depicted in Fig. 6(e), where there is a small increase in normalized intensity over time, representative of a majority of cell track ranges shown in Fig. 6(d). The TGFβ1 condition shows a more dynamic range of normalized intensities spanned by individual cells, and Fig. 6(f) provides an individual cell track representative of the cell subpopulation with a more dynamic range [higher in the violin plot distribution, shown in Fig. 6(g)].

**FIG. 7.**
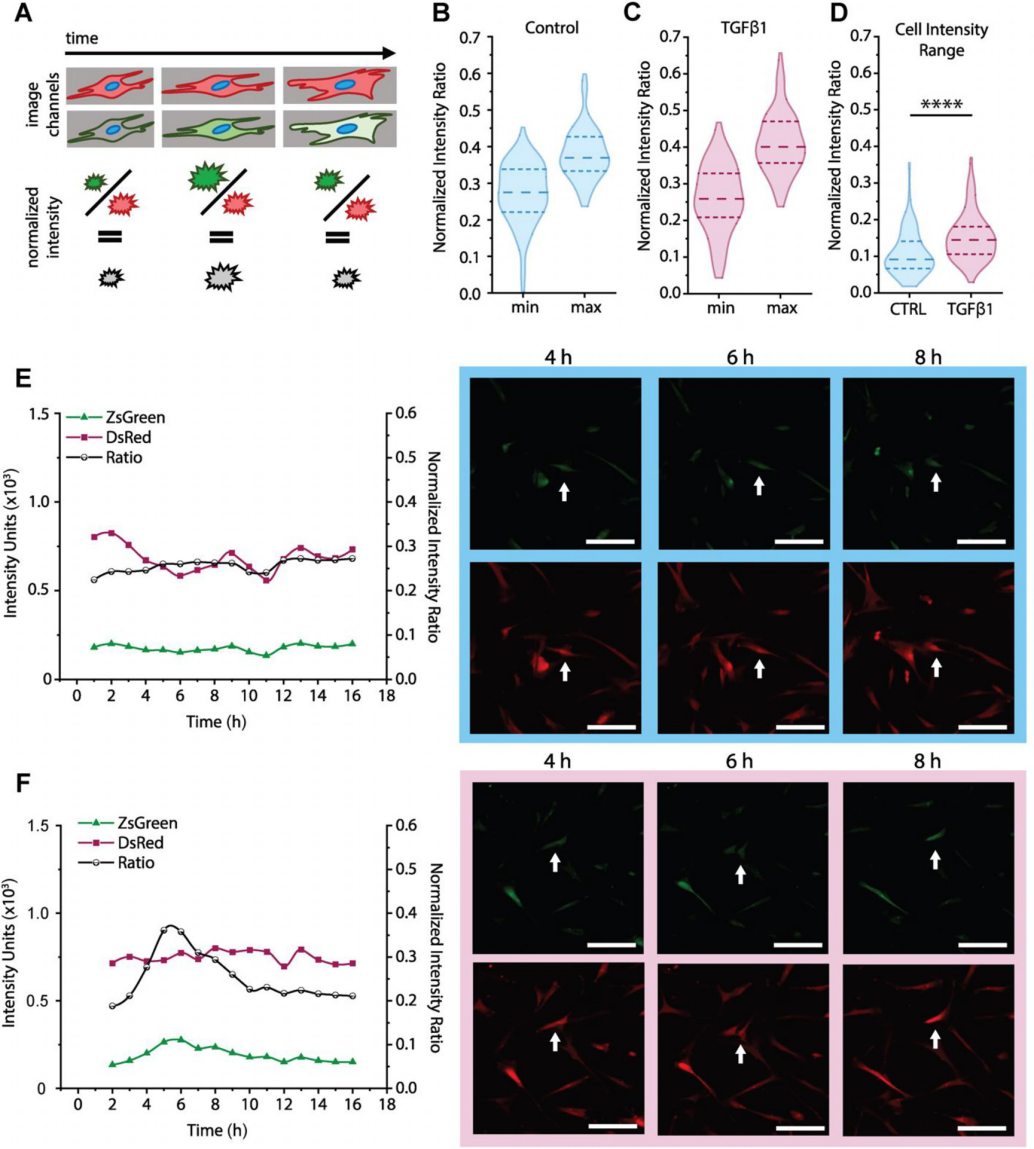
Temporal dynamics of activation in response to biochemical cues: Intermediate reporter. (a) Timecourse image studies identified and quantified individual cell fluorescence intensity values over time and calculated normalized intensity ratios for single cells at each timepoint. (b) Distribution of the minimum and maximum normalized intensity value of each cell over the timecourse (16 h) for control cells. (c) Distribution of the minimum and maximum normalized intensity value of each cell over the timecourse for TGFβ1 treated cells. (d) Distribution of the spread between the maximum and minimum normalized intensity per cell over the timecourse for both control and TGFβ1 treated cells. For each violin plot, >80 cell tracks were analyzed. ^*^p < 0.05, ^**^p < 0.01, ^***^p < 0.001, and ^****^p < 0.0001. (e)–(f) Individual cell tracks of DsRed, ZsGreen, and normalized intensity ratio values over the timecourse from the (e) control and (f) TGFβ1 treated population. White arrows note the tracked cell body. Scale bars are 25 *μ*m.

We conducted the same analysis on the Intermediate reporter live-imaging, where a distinct difference can be seen between minimum and maximum values of the cell dynamic range for both control [Fig. 7(b), Table S12] and TGFβ1 [Fig. 7(c), Table S12] conditions. We chose to only pursue the Intermediate reporter as a representation of transient reporting, as the Fast reporter was challenging to meaningfully capture with imaging. When investigating these further, the TGFβ1 condition shows higher maximum intensity per cell over the timecourse, as compared with the control condition, showing a dynamic response to TGFβ1 treatment [Figs. 7(b), 7(c), and S11]. This difference in dynamics is further validated when comparing the individual cell track dynamic range for both control and treatment conditions: there is a statistically significant increase for individual cells on average in response to TGFβ1, as well as an increase in the width and height of the upper tail of the distribution [Fig. 7(d), Table S12]. Comparing observations of trends in individual cell responses to TGFβ1 between the Stable [Fig. 6(d)] and Intermediate [Fig. 7(d)] reporters, the Intermediate reporter highlights more distinct differences within the cell population generally, whereas the Stable reporter highlights the highest expressing cells most effectively.

We can utilize the Intermediate reporter to investigate individual cell tracks as we did for the Stable reporter, and as would be expected with the destabilized ZsGreen, there is a quantifiable wax and wane of normalized fluorescence intensity in the cell tracks over the timecourse in response to TGFβ1, where Figs. 7(e) and 7(f) show a representative example track from the (**E**) control and (**F**) TGFβ1 conditions, with additional tracks available in Fig. S10. In the TGFβ1 track, a representative accumulation and dissipation of fluorescence intensity demonstrates the timeframe of response for these reporters with faster turnover times, which is well matched for examining rapid cell responses to biochemical cues. For ease of visualizing the timecourse in images, the fluorescence tracks were separated. In these tracks, visual assessment is less directly insightful, and can even suggest that green intensity does not change meaningfully. However, the quantification of normalized fluorescence in these cases reinforces the value of the dual-fluorescence approach for making quantitative comparisons, leading to more robust assessments. Furthermore, the dual-fluorescence approach minimizes some concerns of artifacts inherent to fluorescence microscopy, such as densification of proteins increasing signal.

While we see clear differences in response to TGFβ1 with both Stable and Intermediate reporters, these differences appear somewhat modest. In image analysis, cell tracks that were less than 13 h long were excluded from consideration to ensure proper investigation of cell changes over the timecourse, which may unintentionally bias the data to exclude more actively proliferating cells. For example, this excluded population may be more responsive to biochemical cues, and future studies should take care to robustly include branched daughter cells to more effectively capture the entire cell population. For investigations of utility, these longer tracks were sufficient in demonstrating that these reporters are effective at identifying changes in αSMA transcription in response to biochemical cues.

With this approach now established, future opportunities exist for quantification of timecourse studies for many single cells for insight into individual, subpopulation, and total population responses, specifically in quantifying the rate of ZsGreen accumulation, dissipation, and timeframe of upregulation in response to different biochemical microenvironments. These analyses will be particularly impactful in heterogeneous cell populations (e.g., primary cells), highlighting ample opportunities for a myriad of investigations where this reporter system would have utility for probing temporal dynamics on a single cell basis.

### F. Reporter approach is translatable to primary human cell sources

The techniques for characterization and application of these reporters were established in well characterized human cell lines. With this robust tool kit, this lentiviral reporter approach can be translated into primary cell sources with high efficiency. Normal human lung fibroblasts (NHLF, Lonza) were transduced with the Stable reporter lentivirus at multiplicities of infection (MOI) of 1, 3, and 6, based on functional titers calculated using the model cell line NIH3T3. Briefly, MOI represents the average number of viral copies delivered to each cell during transduction. Fibroblasts then were expanded until approximately 90% confluence and characterized for transduction efficiency with flow cytometry.

**FIG. 8.**
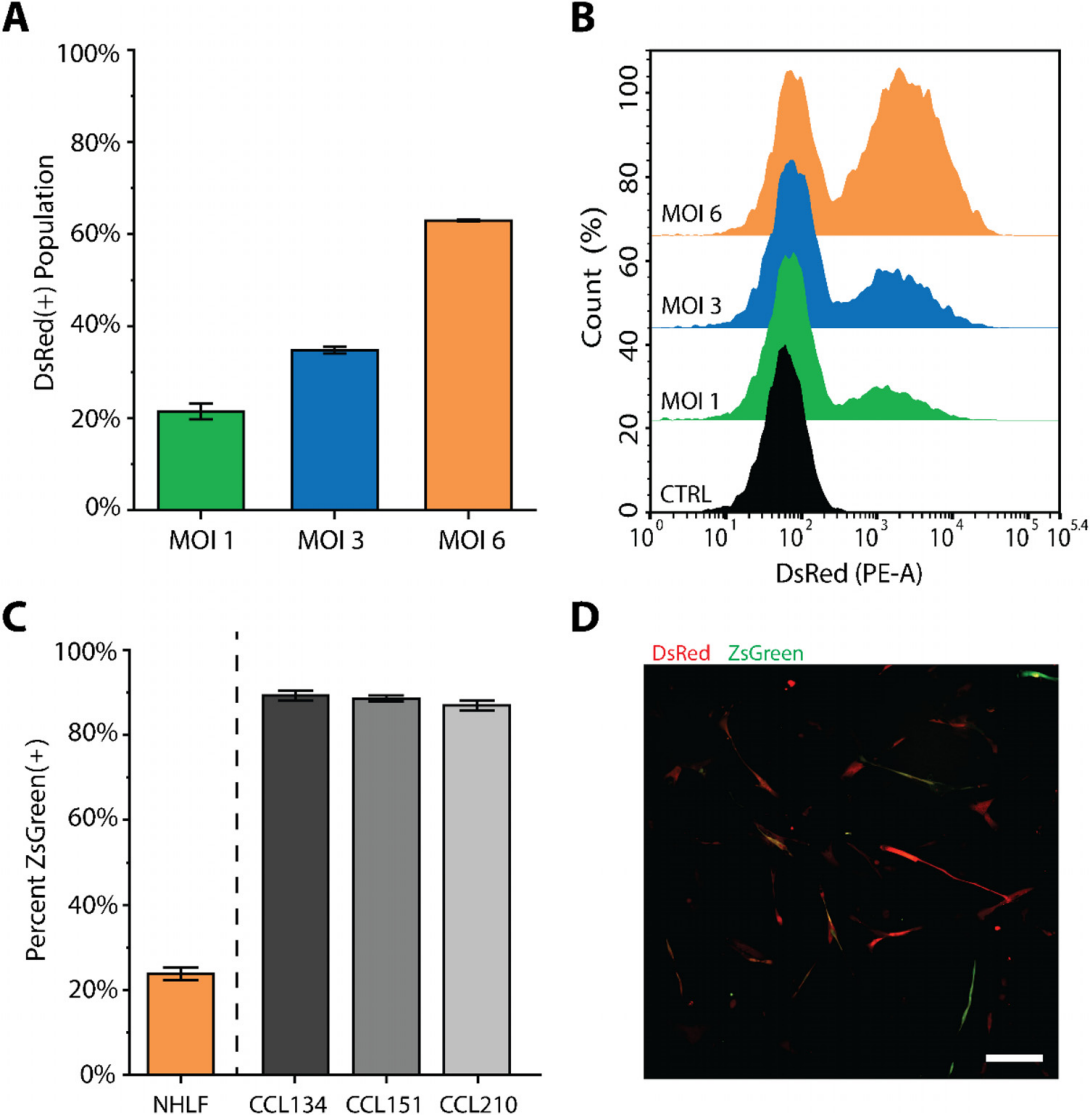
Transduction of primary cells with Stable reporter lentivirus. (a) Population percentage of transduced NHLFs expressing DsRed, indicating successful transduction. Error bars depict the standard deviation of 3 technical replicates. (b) Flow cytometry histogram shifts of NHLFs after transduction. (c) Comparison of NHLF (MOI 6) ZsGreen(+) population and cell lines (data previously shown in Fig. 3). Error bars represent the standard deviation of n ≥ 3 technical replicates, with >3 biological replicates for cell lines and 1 biological replicate for primary NHLFs. (d) Fluorescence imaging of MOI 6 NHLFs at day 4 after transduction. Scale bars are 200 *μ*m.

Notably, all tested MOIs resulted in successful transduction, with approximately 22%, 37%, and 65% of the population positively expressing DsRed fluorescence after 4 days [Figs. 8(a) and 8(b)], demonstrating the efficacy of the lentiviral approach given the known challenges in transducing human primary cells.^36–39^ When compared with the cell line equivalents, the transduced MOI 6 NHLFs demonstrated a much lower population fraction expressing ZsGreen fluorescence, at approximately 23% ZsGreen(+) within the transduced population [DsRed(+)] [Fig. 8(c)]. This observation of differences in ZsGreen expression between primary cells and the cell lines likely is the result of longer culture time of the cell lines on TCPS relative to the primary cells, demonstrating the impact of mechanical memory on αSMA expression. With successful lentiviral transduction on primary human cells, in future studies, investigations can be performed to examine the real-time response of fibroblasts from patient-derived cell sources to fibrotic cues, which is important for the development of new and relevant therapeutic strategies for treating human fibrotic diseases, as well as benchmark observations of the dynamics of αSMA expression provided with the reporter vs traditional assays (e.g., immunostaining, Western blot). These early results suggest the potential utility of this lentiviral reporter system in primary cells, where one can now assess the dynamics of cell response on a single cell basis to more effectively characterize heterogeneous primary cell populations.

## III. SUMMARY AND CONSIDERATIONS FOR APPLICATIONS OF THE REPORTER SYSTEMS

In this work, we established methods for assessing active protein transcriptional processes of αSMA as an indicator of myofibroblast activation in response to microenvironmental cues. We demonstrated the utility of a toolset of reporters, where three different temporal stabilities enable the investigation of protein expression on a range of timescales relevant to different cellular processes. To maximize the utility of such a toolbox, it is helpful to consider which system is most useful for each type of investigation.

We found that the Stable reporter, with its turnover on the order of days, is useful for looking at cellular processes that span a similar timeframe. In this work, the Stable reporter highlighted population shifts and the existence of subpopulations in response to a substrate modulus over multiple passages, and when compared with immunostaining results, demonstrated that stress fiber-positive cells correlated with higher normalized intensity values of the Stable reporter. The Intermediate reporter better captured responses to biochemical cues, where the anticipated timeframe of relevance is on the order of hours. Intermediate and Fast both give insight into responses on the timescale of hours, though with different half-lives. For cells with different metabolic rates, one of these reporters, Intermediate or Fast, may be more effective than the other for adequate detection of accumulated ZsGreen protein. For example, CHO cells with the Fast reporter derivative of ZsGreen demonstrated a protein half-life of 1 h, whereas the same derivative in hMSCs demonstrated a half-life closer to 8–12 h. Furthermore, in considering investigations such as the density comparison that probed cell–cell interactions, the Intermediate and Fast reporters used in tandem showed disparate trends, suggesting that there is potential to illuminate subpopulations using Intermediate and Fast in tandem.

When considering the use of these reporters for investigating differences between cell types, it is important to consider that different cell types may have varying metabolic rates or promoter activation, so reporters are the most useful for assessing differences between (1) different conditions experienced by the same cell type, (2) subpopulations within the same cell sample, and (3) dynamic ranges of sample populations tracked over time. They are less reliable in comparing the absolute values of normalized intensity between cell types, though relative comparisons can still be considered and provide critical insight.

## IV. CONCLUSIONS AND FUTURE DIRECTIONS

In this work, we established the use of lentiviral reporters with different temporal stabilities in human fibroblast populations to track the dynamic expression of αSMA for probing fibroblast activation, where the varied temporal stabilities will enable the assessment of transient transcription processes in response to microenvironmental cues. We established methods for assessing the extent of αSMA expression on a single cell basis and benchmarked vs traditional immunostaining, where we found that stress fiber expressing subpopulations correlate with higher normalized intensity reporter values. We then assessed the reporter cells response to changes in culture substrate stiffness. While the population fraction expressing ZsGreen in many of these conditions was similar, we discovered that the normalized intensity values on a single cell basis provided additional resolution and revealed subpopulations that decreased in expression intensity after sustained culture on soft culture surfaces. Furthermore, we establish imaging methods for assessing cell response to activating cytokines, where we examined individual cell fates over a 16-h timecourse and assessed dynamic ranges of αSMA expression, observing that these reporters can identify small subpopulations of highly activated cells. Finally, we translated this lentiviral method to primary human lung fibroblasts with high efficiency. These innovative methods for assessing fibroblast activation dynamics open the door for future mechanistic studies and the development of more effective therapeutics, as well as provide a general approach for the assessment of expression dynamics of many other proteins.

Many tools for examining fibroblast activation are limited to lower resolution end point assays, or live-cell reporting in cell lines or animal cells that do not fully recapitulate human disease. Live-cell reporting techniques to date have also been used in an on/off fashion, highlighting an opportunity for expanding tools to provide a dynamic range on a single cell basis. With the dual-fluorescence design and range of temporal stabilities implemented here, this toolset of reporters provides dynamic range assessment for single cells and provides temporal tracking capable of reporting on cell response to many activating stimuli. Additionally, the lentiviral-based approach utilized by these reporters opens the door for the tracking of primary human cells sourced from patients for insight into the specifics of human lung fibrosis disease progression. While in this study we show continued cell health and proliferative capacity after transduction (Fig. S2), care must be taken generally when implementing lentiviral approaches to ensure cell function after transduction, as random integration can impact cell function and induce senescence. In these cases, alternative integration strategies, such as CRISPR, can be employed to mitigate these challenges, which have been used to create rat lung fibroblast reporter.^20^ Broadly, our new approach complements the existing ones: for example, (i) transient transfection of fibroblasts with αSMA-luciferase plasmid for a firefly luciferase-driven αSMA gene expression reporter assay, providing insight into responses of the entire population at desired end points over several days in culture with the robust signal-to-noise provided by the luciferase assay;^40^ (ii) use of fibroblasts harvested from transgenic animal models engineered to express a fluorescent protein when proteins associated with fibroblast activation like αSMA are expressed, providing qualitative to semi-quantitative (e.g., percentage of αSMA-positive cells) real-time indicators of protein transcription with non-human cells,^19^ and CRISPR/Cas9 to establish fibroblasts that fluorescently report on αSMA expression with site-specific gene integration,^20^ a useful alternative to lentiviral transduction as noted above. In all, future studies can capitalize on the live-cell tracking capability of the reporters established here for examining single cell and subpopulation response dynamics (e.g., αSMA expression with ZsGreen/DsRed ratio in addition to the tracking of cell motility with DsRed) enabled in more complex, 3D hydrogel systems that capture more aspects of human disease. Furthermore, while the presented studies focused on the establishment of a reporter for αSMA gene expression and thus probed responses to stimuli on the timescale of days,^40–42^ future studies can use the reporter toolset to investigate longer time cellular responses to a range of microenvironment cues.

The studies shown in this work demonstrated that, even in populations with nearly all cells expressing αSMA, subpopulations of fibroblasts with higher αSMA expression can be identified with this reporter approach. This highlights a future opportunity for fluorescence-activated cell sorting (FACS) on these high expressing subpopulations for further analysis, either by isolated culture or in conjunction with cutting-edge techniques, such as single cell RNA sequencing for an in-depth snapshot of the gene expression landscape for these critical cells.

More broadly, this system with internal normalization may prove useful for the investigation of many proteins of interest for a broad range of disease investigations. For example, the αSMA promoter could be substituted with another promoter of interest, highlighting the versatility and modularity of such a reporter approach. Overall, fluorescent reporting systems that enable quantification have a broad range of applications for the investigation of many biological processes and diseases, with the opportunity for tailored, patient-specific hypothesis testing. Furthermore, the application of these systems within *in vitro* model system research will help refine our understanding of human disease dynamics toward more effective therapeutic targeting and design.

## V. METHODS

### A. General cell culture

Human lung fibroblast lines were purchased from ATCC (CCL151 male healthy, CCL134 female IPF, CCL210 female healthy). Primary normal human lung fibroblasts (NHLFs, 52-year-old male, Caucasian) were purchased from Lonza. CCL151 and CCL134 cells were cultured in Ham's F12K medium supplemented with 10% fetal bovine serum (Gibco), 1% penicillin/streptomycin (P/S, Gibco), and 0.2% amphotericin B/Fungizone (Fz, Gibco). CCL210 cells were cultured in MEM (Gibco, cat. 11090081) supplemented with with 10% fetal bovine serum (Gibco), 1% P/S, 0.2% Fz, 1X non-essential amino acids (Gibco, cat. 11140050), 1X GlutaMAX (Gibco, cat. 35050061), and 1X sodium pyruvate (Gibco, cat. 11360070). All cell lines were utilized between passages 7 and 12 and split at less than 90% confluency. Primary NHLFs were cultured in DMEM (Corning, 10–013-CV) supplemented with 10% FBS, 1% P/S, and 0.2% Fz, utilized between passages 5 and 9, and split at 90% confluency. All experiments were conducted with at least three replicates, as detailed in the subsections below, and repeated to ensure reproducibility.

### B. Immunostaining

To fix samples for immunostaining, cells were washed twice with PBS, then fixed with 4% paraformaldehyde solution (prepared from 16% v/v solution purchased from Sigma Aldrich, frozen aliquots) in PBS at room temperature for 10 min. Paraformaldehyde was removed, and then cells were washed twice with PBS. Wells were blocked and cells were permeabilized with a solution of 5 wt. % bovine serum albumin (BSA) and 0.1% v/v Triton X-100 in PBS at room temperature for 1 h. Fixed samples were then stained overnight at 4 °C with a solution of primary mouse anti-alpha smooth muscle actin antibody (Abcam, ab7817) diluted 1:100 into PBS with 2.5 wt. % BSA and 0.05% v/v Triton X-100. Cells were washed three times for 5 min each with the 2.5 wt. % BSA/0.05% v/v Triton X-100 solution. Cells were then stained for 2 h at room temperature with secondary Alexa Fluor 647 goat anti-mouse antibody diluted 1:250 into PBS with 5 wt. % BSA/0.1% v/v Triton X-100. Antibody solution was removed, and cells were washed three times for 5 min each with PBS and then stained with 40 μM Hoechst 33342 (Thermo Scientific) in PBS for 20 min. Samples were washed three times with PBS and stored in PBS in the dark at 4 °C until imaging.

### C. Confocal imaging

Fixed and immunostained samples were imaged on the Zeiss Cell Discoverer 7 microscope using wide-field LEDs at 10X magnification (PlanApochromat 20X/0.95 and 0.5X Optivar) tile scans. For each condition, 3 wells were imaged, with at least 100 cells captured per well. Files were converted to 16-bit TIFFs for analysis. For example, for the immunostained images in figures, brightness was adjusted to improve visualization, whereas raw values for all images were utilized for intensity quantification. Brightness was similarly adjusted for both Intermediate and Fast reporter images for visualization.

### D. Immunostaining image quantification

Images of cells immunostained for αSMA were analyzed using Imaris (version 10, Oxford Instruments). Cell objects were identified by DsRed fluorescence to determine cell bodies, and any clustered cell objects based on Hoechst-stained nuclei were separated. Since DsRed was utilized to determine the cell body, all identified cell objects were considered positively transduced. Identified cell objects were also visually assessed for proper identification, and those with incorrectly identified boundaries that could not be resolved (i.e., cell clusters) were excluded from analysis. For each cell object, mean fluorescence intensities of DsRed, ZsGreen, and AF647 were calculated and exported. Cells were considered αSMA positive if the mean AF647 intensity from αSMA immunostaining of the cell object was above the background AF647 intensity (determined by the average of five AF647 intensity measurements of image background). Stress fibers (identifiable at the captured image resolution) were visually assessed, and a cell object was considered to contain stress fibers if 2 or more distinct fibers were identifiable within the cell body. Stress fiber identification was assessed per cell object to allow for correlation with normalized reporter intensity values. Intensity distributions of cell subpopulations with or without observable stress fibers were compared using Student's t-test with Welch correction for unequal variances, and p-values less than 0.05 were considered significant. Experiments utilized n = 3 replicate wells, with more than 50 cells characterized per well.

### E. Flow cytometry sample preparation

Cells were washed with PBS and trypsinized using 0.25% trypsin and 2.21 mM EDTA solution for 5–7 min at 37 °C. Trypsinization was neutralized with complete media, then the cell suspension was collected and centrifuged for 5 min at 200xg. The cells were washed with PBS and centrifuged, then resuspended in 4% paraformaldehyde solution in PBS at room temperature and fixed for 10 min. After fixation, cells were washed twice with PBS and resuspended in PBS for analysis. Each cell sample was filtered through the cap of a filter top FACS tube and stored at 4 °C or on ice until assessment on BD FACSAria II flow cytometer.

### F. Examination of cell density effects

CCL151, CCL134, and CCL210 fibroblasts of all reporter types and non-reporter controls were seeded at 5000 cells/cm^2^, 10 000 cells/cm^2^, or 20 000 cells/cm^2^ onto tissue culture polystyrene (TCPS) 6-well plates (n = 3 technical replicates, experiment was completed at least twice). Cells were then collected at either 48 h (all other cell densities) or 96 h (5000 cells/cm^2^ condition only), where the 96 h timepoint achieved a similar final density to the 48 h timepoint at 20 000 cell/cm^2^ initial density. Collected cells were fixed for flow cytometry assessment and run on a BD FASCAria II. All seeding conditions were assessed with one-way ANOVA and Tukey's post-hoc analysis within each cell line and reporter type, and p-values below 0.05 were considered significant.

### G. ZsGreen(+) fraction determination of flow cytometry samples

Cell population gate was identified on a density plot of forward scatter area (FSC-A) vs side scatter area (SSC-A), and singlets were identified and gated on a density plot of SSC-H (height) vs SSC-A to identify and remove clusters. DsRed and ZsGreen intensities were plotted as histograms, and gates for DsRed(+) and ZsGreen(+) were determined individually by a control, untransduced sample of the same cell line origin (e.g., untransduced CCL151 cells were used as the CCL151 reporter control), where the gate boundary was determined to be where 0.5%–0.7% of control cells were in the fluorescent protein-positive population. The ZsGreen(+) fraction in bar graph assessments represent the percent of DsRed(+) events that are also positive for ZsGreen.

### H. Mechanical memory assessment

The CCL151 fibroblasts of all reporter types (Stable, Intermediate, and Fast) were seeded at 5000 cells/cm^2^ on either collagen I coated polyacrylamide with a Young's modulus of 2 kPa or 50 kPa (Matrigen) or on collagen I coated TCPS. After 4 days of culture, cells achieved approximately 90% confluency, were trypsinized (washed twice with PBS, 0.25% trypsin, 2.21 mM EDTA, 5–7 mins at 37 °C), and passaged onto fresh plates of the same substrate type at the same density of 5000 cell/cm^2^. Excess cells after seeding were fixed for flow cytometry assessment (n = 3 per condition, per timepoint). Cells seeded for passage 2 were allowed to grow for 4 days to approximately 90% confluency and were trypsinized and fixed for flow cytometry assessment. Cells were fed every 2 days. The impact of culture substrate and passage number on ZsGreen(+) population fraction was assessed within each reporter type by two-way ANOVA with Tukey's post-hoc analysis (OriginLabs). For distribution analyses, raw fluorescence intensity values for each cell in the ZsGreen(+) gate were exported from the flow analysis software (NovoExpress), and a normalized intensity ratio of ZsGreen divided by DsRed was calculated. These raw distributions were heavily right skewed, and to correct these distributions to approach normality and improve visualization of the distribution shifts between conditions, a box-cox transformation was performed (λ = 0.075) across a composite dataset of all conditions to determine the optimized lambda value (JMP). These distributions (kernel smoother) were plotted for comparison across conditions, and shifts were qualitatively assessed.

### I. Time-lapse imaging

CCL151-Stable and CCL151-Intermediate reporter cells were cultured in T-75 flasks in F12K complete medium with 10% FBS, 1% penicillin/streptomycin, and 0.2% amphotericin B. Cells were trypsinized for seeding, centrifuged, and resuspended in media supplemented with 1% FBS to minimize any influence of FBS on fibroblast activation. Cells were seeded in glass bottomed plates at 5000 cells/cm^2^ and cultured for 24 h. At 24 h, cells were fed with either fresh media (control) or fresh media containing 10 ng/mL TGFβ1 (Peprotech, human, HEK293T derived), and samples were imaged on the Zeiss Cell Discoverer 7 confocal microscope for ∼16 h. Images were taken using wide-field LEDs at 10X magnification (20X/0.95 and 0.5X Optivar) using tile scans at 1 h intervals. Timelapses were exported as 16-bit TIFFs and analyzed on Imaris.

### J. Imaris cell tracking analysis

Timelapses of CCL151 reporter cells treated with either 0 ng/mL or 10 ng/mL TGFβ1 were imported into Imaris analysis software. Cell objects were identified by DsRed fluorescence intensity, and cells were separated by seed point. Since cells were identified by DsRed channel intensity, all cell objects were assumed to be positively transduced by reporter cells. “Cell tracks,” which normalize fluorescence intensity within individual cells over time, were determined by the software, and tracks with 13 or more timepoints were considered for analysis. For all conditions assessed, >80 tracks were analyzed. For each cell object, the mean fluorescence intensity of DsRed and ZsGreen was exported, and a normalized intensity of ZsGreen/DsRed was calculated for each cell track over time. To summarize changes in the population over time, each cell was assessed for its maximum and minimum normalized intensity across all recorded timepoints, and the dynamic range of intensities spanning over the timecourse was also calculated. These values (maximum, minimum, and range) were plotted as functions of population for each condition assessed (control and TGFβ1 treated, Stable and Intermediate reporters). Statistical analysis between control and TGFβ1 conditions for minima, maxima, and ranges was assessed by Student's t-test, and p-values less than 0.05 were considered significant.

## SUPPLEMENTARY MATERIAL

See the supplementary material for additional materials and methods and supplementary figures and tables. Supplementary figures include data supporting transduced cell line function, images and videos used for assessing the half-life of destabilized reporter cell lines, the sequence of the intermediate reporter, comparison between alpha smooth muscle actin immunostaining and Intermediate and Fast reporters, and additional traces of reporter cells imaged over time with and without TGFβ1 treatment. Supplementary tables include exponential coefficients from cell trace fits of the Intermediate CCL151 reporter line for quantitative half-life determination, as well as full results of the statistical analyses from various experiments.

## Data Availability

The data that support the findings of this study are available from the corresponding author upon reasonable request.
